# Adverse impact of elevated serum progesterone and luteinizing hormone levels on the hCG trigger day on clinical pregnancy outcomes of modified natural frozen-thawed embryo transfer cycles

**DOI:** 10.3389/fendo.2022.1000047

**Published:** 2022-12-01

**Authors:** Chenyang Huang, Xiaoyue Shen, Qingqing Shi, Huizhi Shan, Yuan Yan, Jingyu Liu, Na Kong

**Affiliations:** ^1^ Center for Reproductive Medicine and Obstetrics and Gynecology, Drum Tower Clinic Medical College of Nanjing Medical University, Nanjing, China; ^2^ Reproductive Medicine Center, Nanjing Drum Tower Hospital, The Affiliated Hospital of Nanjing University Medical School, Nanjing, China; ^3^ Center for Molecular Reproductive Medicine, Nanjing University, Nanjing, China

**Keywords:** modified natural cycle, frozen-thawed embryo transfer, serum progesterone level, serum luteinizing hormone level, clinical pregnancy rate, live birth rate

## Abstract

**Research question:**

The relationship between serum progesterone (P) and luteinizing hormone (LH) levels on the human chorionic gonadotropin (hCG) trigger day and the clinical pregnancy outcomes in modified natural frozen-thawed embryo transfer (mNC-FET) cycles are controversial.

**Design:**

This was a retrospective study of 788 mNC-FET cycles. A smooth fitting curve and threshold effect analysis was performed to identify the effect of serum P and LH levels measured on the hCG day on the clinical pregnancy rate (CPR) and live birth rate (LBR) of mNC-FET cycles.

**Results:**

The CPR and LBR decreased significantly when the LH level on the hCG day was greater than or equal to 32 IU/L. Further subgroup analysis showed that the CPR decreased significantly when the P level on the hCG day was equal to or greater than 1 ng/mL. When the P level was lower (< 1 ng/mL), the patients with an LH level greater than or equal to 32 IU/L had reduced CPR and LBR in mNC-FET cycles.

**Conclusion:**

Applying the hCG trigger on a day with a higher P level (≥ 1 ng/mL) leads to a decreased CPR and LBR. hCG administration with a higher LH level (≥ 32 IU/L) also leads to a decreased CPR and LBR in mNC-FET cycles when the P level is less than 1 ng/mL.

## Introduction

With improvements in assisted reproduction and embryo cryopreservation technology, the proportion of frozen-thawed embryo transfer (FET) cycles has increased over the years (1). There are some endometrial preparation protocols for FET cycles, such as hormone replacement therapy (HRT), natural cycles (NC) and modified natural cycles (mNC) (2, 3). Since the natural cycles and modified natural cycles do not require ovarian stimulation and meet the physiological requirements of embryo implantation, they can avoid the endometrial synchronization disorder caused by superphysiological levels of oestrogen and have become the most common endometrial preparation protocols (4, 5). In modified natural cycles, dominant follicle development is detected by ultrasound and changes in hormone levels are measured to detect the human chorionic gonadotropin (hCG) trigger time, which is especially critical for the correct transformation of the endometrium (6, 7).

A study by Weissman et al. indicated that an elevated progesterone (P) level might occur 12 hours before the LH surge that triggers ovulation (8). Therefore, an increase in P levels may occur during the hCG trigger. Progesterone is known to act on endometrial cells, regulate endometrial receptivity and induce the window of implantation (9, 10). Many studies have shown that after controlled ovarian hyperstimulation (COH), an increased P level may affect pregnancy outcomes in fresh embryo transfer cycles (11–13). On the other hand, studies have also shown that abnormal luteinizing hormone (LH) levels will lead to poor endometrial hyperplasia and affect pregnancy outcomes (14–16). However, the effects of P and LH levels on pregnancy outcomes in the mNC-thawed embryo transfer (mNC-FET) remain controversial (6, 17, 18). Therefore, we conducted a retrospective study of the mNC-FET cycles in our reproductive medicine centre at Nanjing Drum Tower Hospital to investigate the relationships between serum P and LH levels before the hCG trigger and clinical pregnancy outcomes.

## Materials and methods

### Patient population

From January 2016 to December 2020, 788 modified natural cycle-frozen embryo transfer (mNC-FET) patients at the reproductive medicine centre of Nanjing Drum Tower Hospital were included in this retrospective study. Participants were 20 to 45 years of age when undergoing mNC-FET. The exclusion criteria for this study are listed below: 1. More than three embryo transfer cycles (such as recurrent implantation failure and recurrent miscarriages); 2. Taking hormonal replacement drugs; 3. Patients with endometriosis (EMS) or adenomyosis; 4. Patients with hydrosalpinx or endometrial lesions. The characteristics of all of the patients are shown in **Table S1**.

### Procedures

On the 10^th^-12^nd^ day of the patients’ menstrual cycle, follicular development and the endometrium were monitored by ultrasound. Serum oestrogen (E_2_), LH and P levels were measured, and the ultrasound monitoring was repeated depending on the diameter of the dominant follicle. When the follicle diameter is less than 14 mm, we usually measure the hormone levels every 1-2 days and when the follicle diameter is greater than 14 mm, we measure them every day. The E_2_, LH and P concentrations in the seum were determined using electrochemiluminescence kits and a Roche automatic electrochemiluminescence immunoassay system (Cobas e801). All operation steps were carried out in strict accordance with the manufacturer’s instructions. When the dominant follicle diameter was ≥ 16 mm, hCG (5000 IU, Livzon Pharmaceutical) was administered to induce ovulation, along with oral dydrogesterone tablets starting from the 2^nd^ day after the hCG trigger (Duphaston, Abbott, 20 mg b.i.d. × 4 or 6 days) to assist with the endometrial transformation. On the fifth day and the seventh day after the hCG trigger, cleavage-stage embryos or blastocysts were thawed and transferred respectively. Patients usually take Duphaston (20 mg dydrogesterone, b.i.d.) for luteal support, which would be maintained for 2 months after FET of the pregnant patients. At 12-14 days after FET, the serum β-human chorionic gonadotropin (β-hCG) levels were measured. Biochemical pregnancy was determined as a preliminarily increased β-hCG level (> 20 IU/L). To confirm clinical pregnancy, patients with biochemical pregnancy were examined by transvaginal ultrasound 28-30 days after FET. Clinical pregnancy was defined as the presence of a gestational sac. The patient was continuously followed up to identify any abnormalities in the pregnancy.

### Statistical analysis

We performed a smooth curve fit analysis, which applied a generalized additive model (GAM) to investigate the relationship between the P or LH levels before the hCG trigger and the CPR or LBR of mNC-FET cycles. In addition, threshold and saturation effect analyses were conducted to detect the potential cut-off value of the serum P and LH levels on the hCG day. After testing for normality by the Kolmogorov-Smirnov test, a t-test was used for the normally distributed variables and the Mann-Whitney U test was used for the the nonnormally distributed variables. Furthermore, the chi-squared test was employed for the categorical variables meeting the test criteria (theoretical frequency (T) > 5 and sample number (n) > 40). All variables are presented as the mean ± standard deviation (SD). The two-sided α level was set at 0.05. All statistical analyses were performed using EmpowerStats (www.empowerstats.com, X&Y solutions, Inc. Boston MA) and R software version 3.6.0 (http://www.r-project.org).

### Ethics

This study received ethical approval from the ethics committee of Nanjing Drum Tower Hospital.

## Results

**Figure 1:**
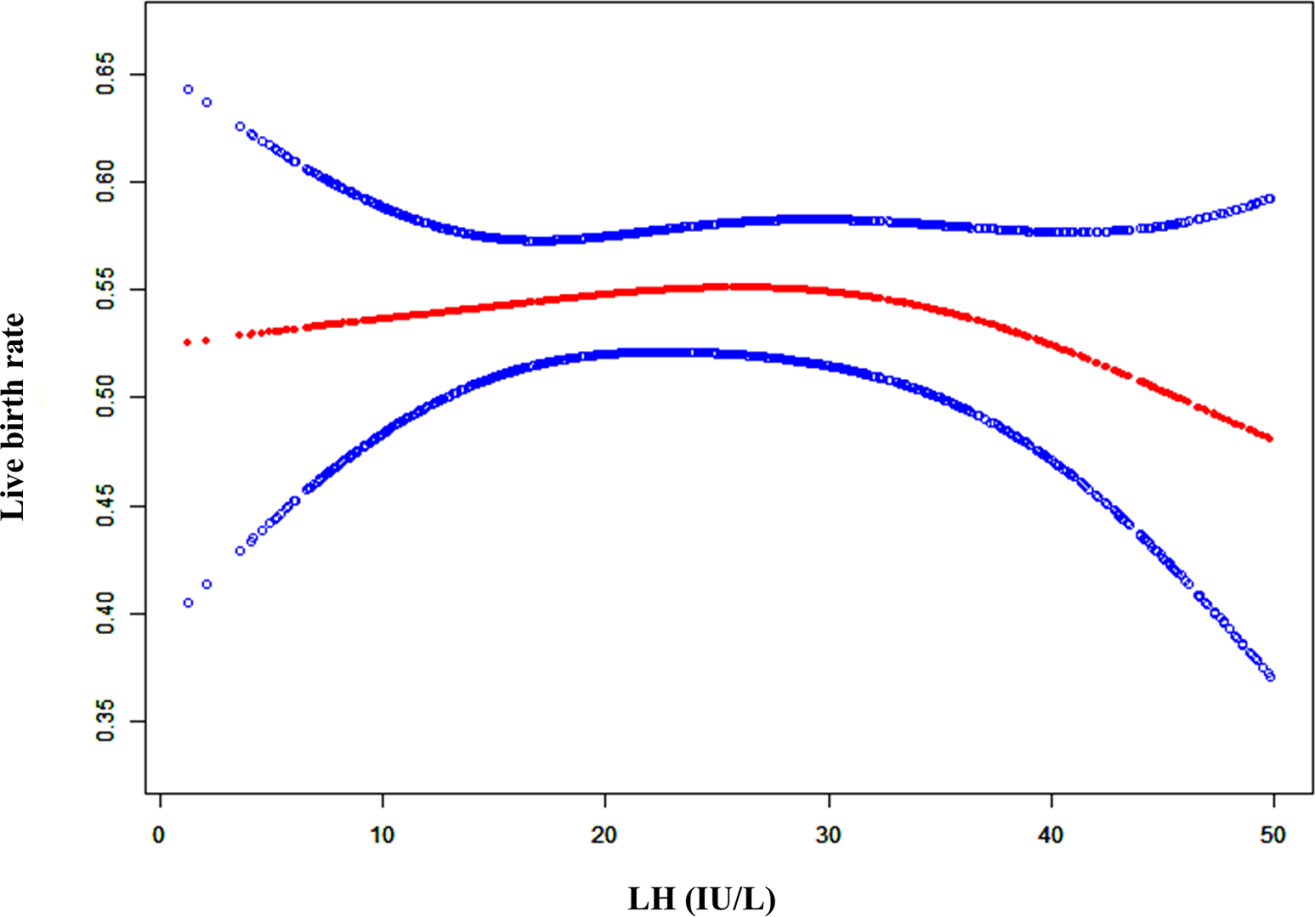
** **A smooth fitting curve analysis of the relationship between LH levels on the hCG day and the live birth rates. The live birth rate of the patients decreased as the LH level on the hCG day gradually increased when the LH level was beyond a certain value.

As shown in **Figure S1** and **Figure 1**, when the LH level on the hCG day was lower, the clinical pregnancy rate (CPR) and live birth rate (LBR) was stable. However, the CPR and LBR decreased once the serum LH level exceeded a certain degree. To explain the LBR variation trend with the serum LH levels observed in **Figure 1**, threshold effect analysis was conducted, which revealed that there was a curvilinear relationship between the LBR and LH levels (**Table 1**, logarithmic likelihood ratio = 0.047). The LBR decreased as the serum LH level on the hCG day increased when the LH level reached or exceeded 32 IU/L (**Table 1**, aOR: 0.961, 95% CI: 0.925-0.999, p = 0.043). When the LH concentration was lower than 32 IU/L, the LBR was independent of the serum LH level on the hCG day (**Table 1**, aOR: 1.013, 95% CI: 0.993-1.033, p = 0.196).

**Table 1:** Threshold effect analysis of serum LH level (IU/L) at the hCG day on the LBR.

Outcome	Live birth		
Model I (linear)	aOR	95% CI	p value
**Linear effect**	0.997	(0.985, 1.009)	0.647
**Model II (polyline)**	**aOR**	**95% CI**	**p value**
**Predicted threshold (K, LH level, IU/L)**	32		
**Effect 1 (<K)**	1.013	(0.993, 1.033)	0.196
**Effect 2 (>K)**	0.961	(0.925, 0.999)	0.043
**variability of effectiveness**	0.366	(0.900, 0.999)	0.047
**Logarithmic likelihood ratio test**	0.047		

LH, luteinizing hormone; aOR, adjusted odds ratio; CI, confidence interval; K, predicted threshold.

Adjust for: female age, male age, number of transferred embryos, type of transferred embryos and endometrial thickness.

**Figure 2:**
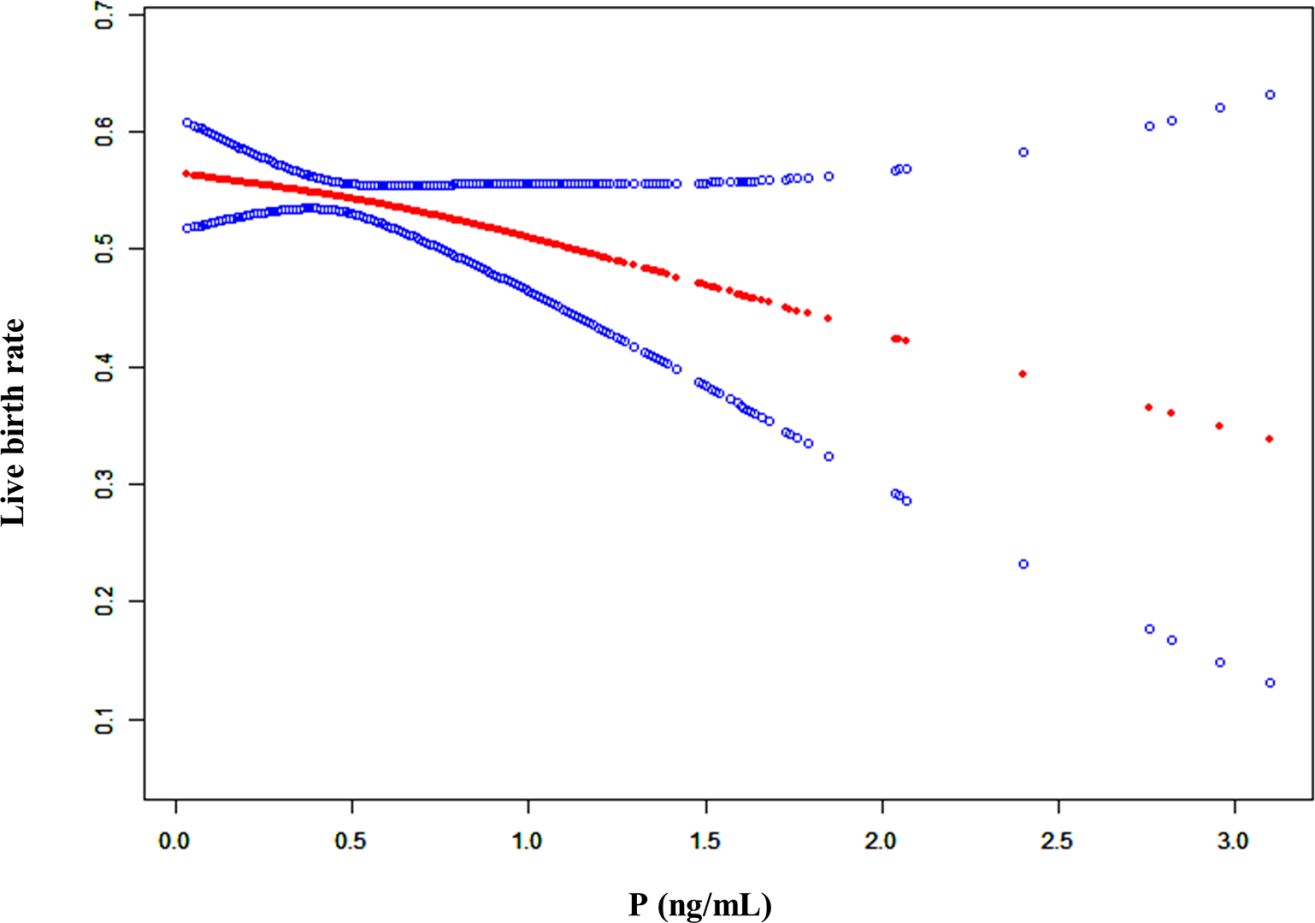
A smooth fitting curve analysis of the relationship between P levels on the hCG day and the live birth rates. The live birth rate of the patients decreased as the P level on the hCG day gradually increased.

In addition, we conducted a smooth curve analysis to evaluate whether the serum P level on the hCG day had any effects on the pregnancy outcomes in mNC-FET cycles, which showed that the CPR and LBR decreased gradually with the increase in serum P level (**Figures 2** and **S2**). A threshold effect analysis was conducted, and results suggested that the LBR was relatively stable when there was a lower serum P level on the hCG day (< 1 ng/mL). When the P level reached or exceeded 1 ng/mL, the LBR decreased (**Table 2**, aOR: 0.558, 95% CI: 0.340-0.916, p = 0.021). In addition, the results of interaction analysis suggested that different levels of LH did not interfere with the adverse effects of elevated P levels on the LBR (**Table S2**).

**Table 2:** Threshold effect analysis of serum P level (ng/mL) at the hCG day on the LBR.

Outcome	Live birth		
Model I (linear)	aOR	95% CI	p value
**Linear effect**	0.812	(0.590, 1.116)	0.199
**Model II (polyline)**	**aOR**	**95% CI**	**p value**
**Predicted threshold (K, P level, ng/mL)**	1.0		
**Effect 1 (<K)**	2.162	(0.789, 5.923)	0.134
**Effect 2 (≥K)**	0.558	(0.340, 0.916)	0.021
**variability of effectiveness**	0.258	(0.068, 0.974)	0.046
**Logarithmic likelihood ratio test**	0.044		

P, progesterone; aOR, adjusted odds ratio; C, confidence interval; K, predicted threshold.

Adjust for: female age, male age, number of transferred embryos, type of transferred embryos and endometrial thickness.

**Figure 3:**
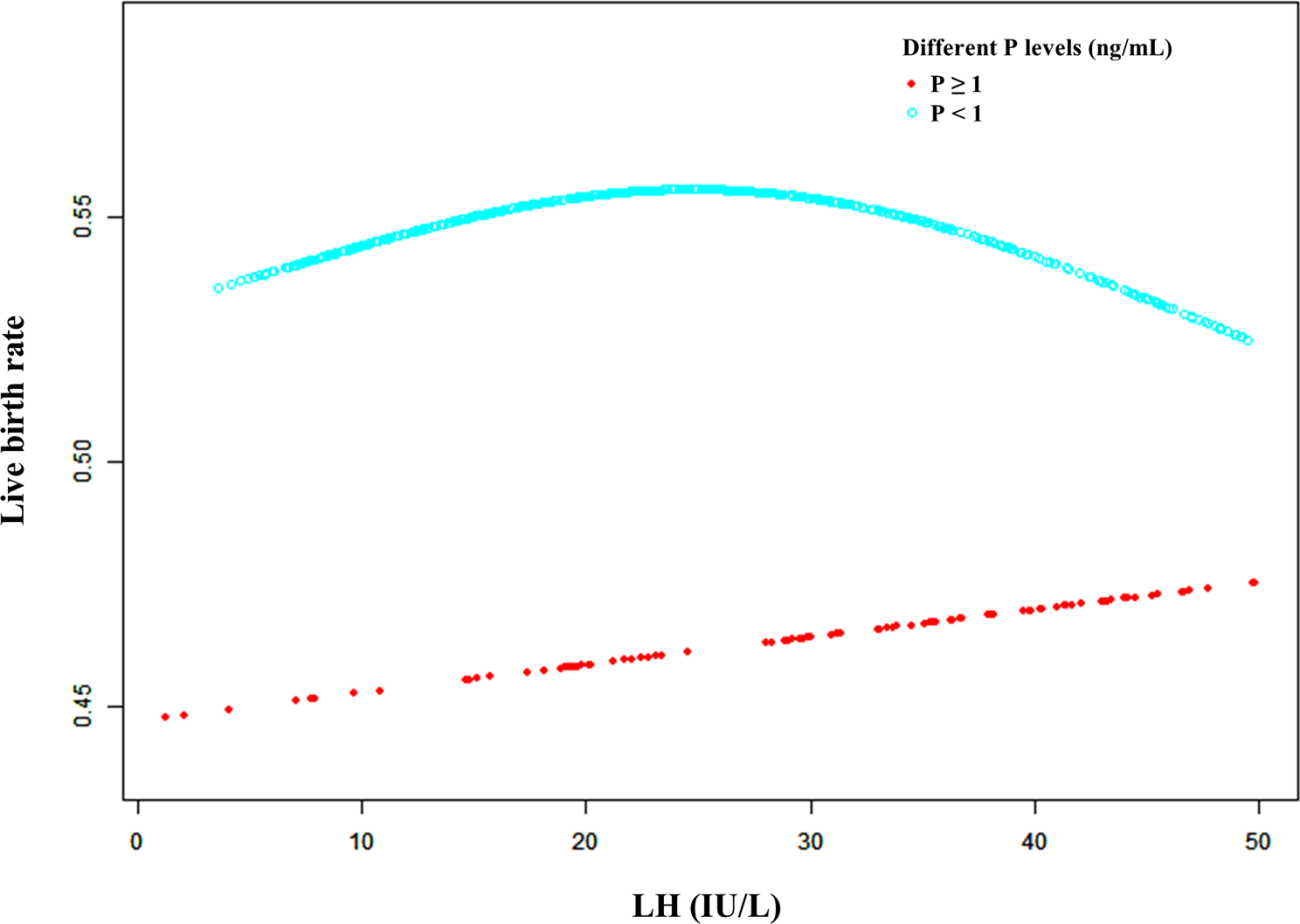
** **A smooth fitting curve analysis of the relationship between LH levels on the hCG day and the live birth rates of patients with different P levels. The live birth rate decreased as the LH level on the hCG day gradually increased when the P level was equal to or greater than 1 ng/mL. In addition, the live birth rate of the patients with a lower P level (less than 1 ng/mL) on the hCG day decreased as the LH level gradually increased when the LH level was beyond a certain value.

Furthermore, a smooth fitting curve was applied for the relationship between serum LH levels on the hCG day and CPR or LBR according to the different serum P levels (≥ 1 ng/mL and < 1 ng/mL) of mNC-FET cycles (**Figures 3** and **S3**). In the higher P level group (≥ 1 ng/mL), a significant downtrend of CPR was observed as the LH levels increased before the hCG trigger, while a slight increase (without statistical significance) of LBR was observed. In addition, when P level was lower (< 1 ng/mL), the relationship between LH level and LBR was similar to that shown in **Figure 1**. Therefore, a threshold effect analysis for the FET cycles with lower serum P levels (< 1 ng/mL) was conducted suggesting that no correlation was detected between the LBR and a lower LH level (< 32 IU/L) (**Table 3**, aOR: 1.014, 95% CI: 0.993-1.035, p = 0.206). However, when the serum LH level was ≥ 32 IU/L, the LBR decreased as the LH level on the hCG day increased (**Table 3**, aOR: 0.957, 95% CI: 0.917-0.999, p = 0.045).

**Table 3:** Threshold effect analysis of serum LH level (IU/L) at the hCG day on the LBR according to differennt serum P levels.

P group	≥ 1			< 1		
Model I (linear)	aOR	95% CI	p value	OR	95% CI	p value
**Linear effect**	1.011	(0.977, 1.045)	0.539	0.996	(0.982, 1.010)	0.564
**Model II (polyline)**	**aOR**	**95% CI**	**p value**	**OR**	**95% CI**	**p value**
**Predicted threshold (K, LH level, IU/L)**	9.6			32.0		
**Effect 1 (<K)**	1.774	(0.750, 4.195)	0.192	1.014	(0.993, 1.035)	0.206
**Effect 2 (>K)**	0.991	(0.953, 1.031)	0.656	0.957	(0.917, 0.999)	0.045
**variability of effectiveness**	0.559	(0.233, 1.340)	0.192	0.944	(0.892, 0.999)	0.049
**Logarithmic likelihood ratio test**	0.051			0.049		

P, progesterone; LH, luteinizing hormone; aOR, adjusted odds ratio; CI, confidence interval; K, predicted threshold.

Adjust for: female age, male age, number of transferred embryos, type of transferred embryos and endometrial thickness.

## Discussion

With the continuous advancement of ART and changes in embryo transfer protocols, such as the application of COH, improvement of embryo cryopreservation and the application of whole embryo freezing, the proportion of FET is increasing (19). The results of a meta-analysis suggested that there were no significant differences among endometrial preparation protocols for FET, and no endometrial preparation regimen was specifically recommended (9). The natural cycle is consistent with the patient’s basic physiological condition, while appropriate endometrial synchronization can be obtained. However, patients undergoing natural cycles need repeated hormone tests and ultrasound monitoring, which are often cancelled due to various uncontrollable factors. An artificial hormone replacement cycle does not require repeated tests and makes it easier to adjust the timing of embryo transfer, but patients need to take longer and higher doses of exogenous oestrogen. However, in a modified natural cycle, by administering exogenous hCG, we can accurately control the timing of induction of endometrial transformation and embryo transfer, which can reduce the cancellation rate of natural cycles.

An increasing number of studies suggest that modified natural cycles are a good endometrial preparation protocol for FET with similar or better pregnancy outcomes than HRT-FET or NC-FET cycles (20–22). Weissman et al. (9) concluded that compared with a natural cycle, a modified natural cycle protocol could improve patients’ compliance and comfort, significantly reduce the number of patients’ cycle cancellations, and lead to acceptable pregnancy outcomes. Huberlant et al. believed that a modified natural cycle could not only achieve better clinical pregnancy outcomes, but also reduce cycle costs (21).

Previous studies in our centre also showed that the clinical pregnancy outcomes of mNC-FET were superior to NC-FET. A study by Arefi et al. (23) showed that modified natural cycles led to better pregnancy outcomes in repeated implantation failure (RIF) patients. However, a study by 7 concluded that the clinical pregnancy outcome of mNC-FET cycles was obviously worse than that of NC-FET cycles, which might be related to the population heterogeneity, the timing of hCG induction and the differences in luteal support protocols (7, 9). Our study showed that the CPR and LBR of the mNC-FET were 61.29% and 53.81%, respectively, suggesting that mNC-FET cycles can obtain good pregnancy outcome.

Some studies have assessed serum P levels before or on the transfer day, but few studies have explored the influence of serum P levels on the hCG day on the clinical outcomes in mNC-FET cycles. Groenewoud et al. focused on the influence of elevated P levels on pregnancy outcomes, including 271 modified natural cycle freeze-thaw embryo transfers (9). Their results showed that the serum P levels of 24.4% of patients were higher than 4.6 nmol/L, but did not significantly affect their live birth rates. A study in 2014 showed that the serum P level increased earlier than the LH surge in 28.4% of patients, but the increase in the serum P level did not affect the clinical pregnancy outcome (24). In this study, our results showed that the CPR decreased significantly with an increase in serum P levels (> 1 ng/mL) on the hCG day in 788 mNC-FET cycles. The window of embryo implantation has a specific limit, which corresponds to the time when the endometrium receives progesterone stimulation. We believe that an early increase in progesterone level may lead to an earlier endometrial receptive period, thus affecting the rate of embryo implantation. Therefore, the difference between our study and those of others may be due to the different time arrangements for embryo transfer. However, at the same time, this difference might also be due to the higher CPR and LBR in our centre which might make it easier to demonstrate the impact of higher P levels on clinical outcomes.

The study of Fatemi et al. (24) suggested that the pregnancy rate of patients with LH greater than 18 IU/L was significantly reduced in mNC-FET cycles. Irani et al. ‘s study showed that serum LH and E_2_ levels should be detected during the natural cycle for accurate embryo transfer timing, which can improve the pregnancy rate of NC-FET cycles (25). At the same time, some studies have explored the correlation between increased LH levels and the timing of hCG injection in mNC-FET cycles, and it is believed that an increased LH level does not necessarily require cancelling the embryo transfer (26). Groenewoud et al. showed that increased LH levels occurred in 44.3% of total cycles, and no difference in the live birth rate was found between the elevated and nonelevated LH groups (27). Our study suggests that when the serum LH level on the hCG day is less than 32 IU/L, the LH level does not affect patients’ clinical pregnancy rates. When the LH level reached or exceeded 32 IU/L, the LBR decreased with increasing LH level.

Most previous studies independently investigated the influences of serum P and LH levels on pregnancy outcomes. Their results suggest that when the serum P level increases (0.8 ng/mL to 3.0 ng/mL), the pregnancy rate after hCG induction decreases (28). Most of the time, the serum concentration of LH fluctuates between 5-20 IU/L, and 24-36 hours before ovulation, the serum LH level starts a surge to 25-40 IU/L (20). Therefore, serum LH and P levels are usually considered together to determine the timing of ovulation, and other studies have suggested that increased serum LH and P levels can affect endometrial receptivity (16, 27).

Therefore, our study innovatively explored the joint effects of serum LH and P levels on the LBR of mNC-FET cycles. The threshold effect analysis of serum P level on the hCG day showed that the LBR decreased significantly when the P level reached or exceeded 1 ng/mL in mNC-FET cycles. The patients were further grouped by their serum P level, and the threshold effect analysis results suggested that when the serum P level on the hCG day was lower (< 1 ng/mL) and the serum LH level was higher (≥ 32 IU/L), the LBR was also significantly reduced. Therefore, our resluts suggest that hCG induction is not recommended when the serum P level is equal to or greater than 1 ng/mL. Additionally, hCG induction is not recommended for patients with lower serum P levels (< 1 ng/mL) and higher LH levels (≥ 32 IU/L). However, this conclusion needs to be confirmed by randomized controlled clinical studies.

This study has some limitations. Our study was limited to unpretreated mNC-FET cycles. In addition, although the number and type of embryos transferred were used as adjustment variables for statistical analysis, specific embryo scores were not included. In addition, due to the limitations of our data system, it was difficult for us to directly obtain the corresponding data of oocyte-pick-up cycles from patients in the FET cycles, so we could not give further analysis of potential influence variables of oocyte-pick-up cycles. Its retrospective design is the main shortcoming of this study. To further clarify the effects of serum LH and P levels before hCG induction on the clinical pregnancy outcomes of mNC-FET cycles, higher quality and large-scale randomized controlled trials are needed. Thus, the timing of hCG induction can be more accurately selected to avoid unnecessary cycle cancellations and maintain a higher clinical pregnancy rate while saving time and costs.

## Conclusions

In conclusion, elevated serum P and LH levels on the hCG day have an impact on CPR and LBR in the mNC-FET cycle. When the serum P level on the hCG day is greater than or equal to 1 ng/mL, the CPR and LBR are significantly reduced. For patients with lower serum P levels (< 1 ng/mL), if the serum LH level is higher (≥ 32 IU/L), the LBR are also significantly reduced.

## Data availability statement

The original contributions presented in the study are included in the article/**Supplementary Material**. Further inquiries can be directed to the corresponding authors.

## Author contributions

CYH, JYL and NK contributed to study design, execution, acquisition, analysis, and interpretation of data, manuscript drafting, and critical discussion. XYS, QQS, HZS and YY contributed to acquisition and interpretation of data, manuscript drafting, and critical discussion. All authors contributed to the article and approved the submitted version.

## Funding

This work was supported by the National Natural Science Foundation of China (81801530, 81601246), Reproductions Research Program of Young and Middle-aged Physicians and China Health Promotion Foundation. There are no conflict of interests.

## Conflict of interest

The authors declare that the research was conducted in the absence of any commercial or financial relationships that could be construed as a potential conflict of interest.

## Publisher’s note

All claims expressed in this article are solely those of the authors and do not necessarily represent those of their affiliated organizations, or those of the publisher, the editors and the reviewers. Any product that may be evaluated in this article, or claim that may be made by its manufacturer, is not guaranteed or endorsed by the publisher.

## Supplementary material

Supplementary Figure 1 A smooth fitting curve analysis of the relationship between LH levels on the hCG day and the clinical pregnancy rates. The clinical pregnancy rate of the patients decreased obviously as the LH level on the hCG day gradually increased when the LH level was beyond a certain value.

Supplementary Figure 2 A smooth fitting curve analysis of the relationship between P levels on the hCG day and the clinical pregnancy rates. The clinical pregnancy rate of the patients decreased obviously as the P level on the hCG day gradually increased.

Supplementary Figure 3 A smooth fitting curve analysis of the relationship between LH levels on the hCG day and the clinical pregnancy rates of patients with different P levels. The clinical pregnancy rate decreased as the LH level on the hCG day gradually increased when the P level was equal to or greater than 1 ng/mL. In addition, the clinical pregnancy rate of the patients with a lower P level (less than 1 ng/mL) on the hCG day decreased as the LH level gradually increased when the LH level was beyond a certain value.
