# Supplementary figures and images for: Adverse impact of elevated serum progesterone and luteinizing hormone levels on the hCG trigger day on clinical pregnancy outcomes of modified natural frozen-thawed embryo transfer cycles
Source: Front Endocrinol (Lausanne). 2022 Dec 1;13:1000047. doi: 10.3389/fendo.2022.1000047 (PMC9751419; doi:10.3389/fendo.2022.1000047)

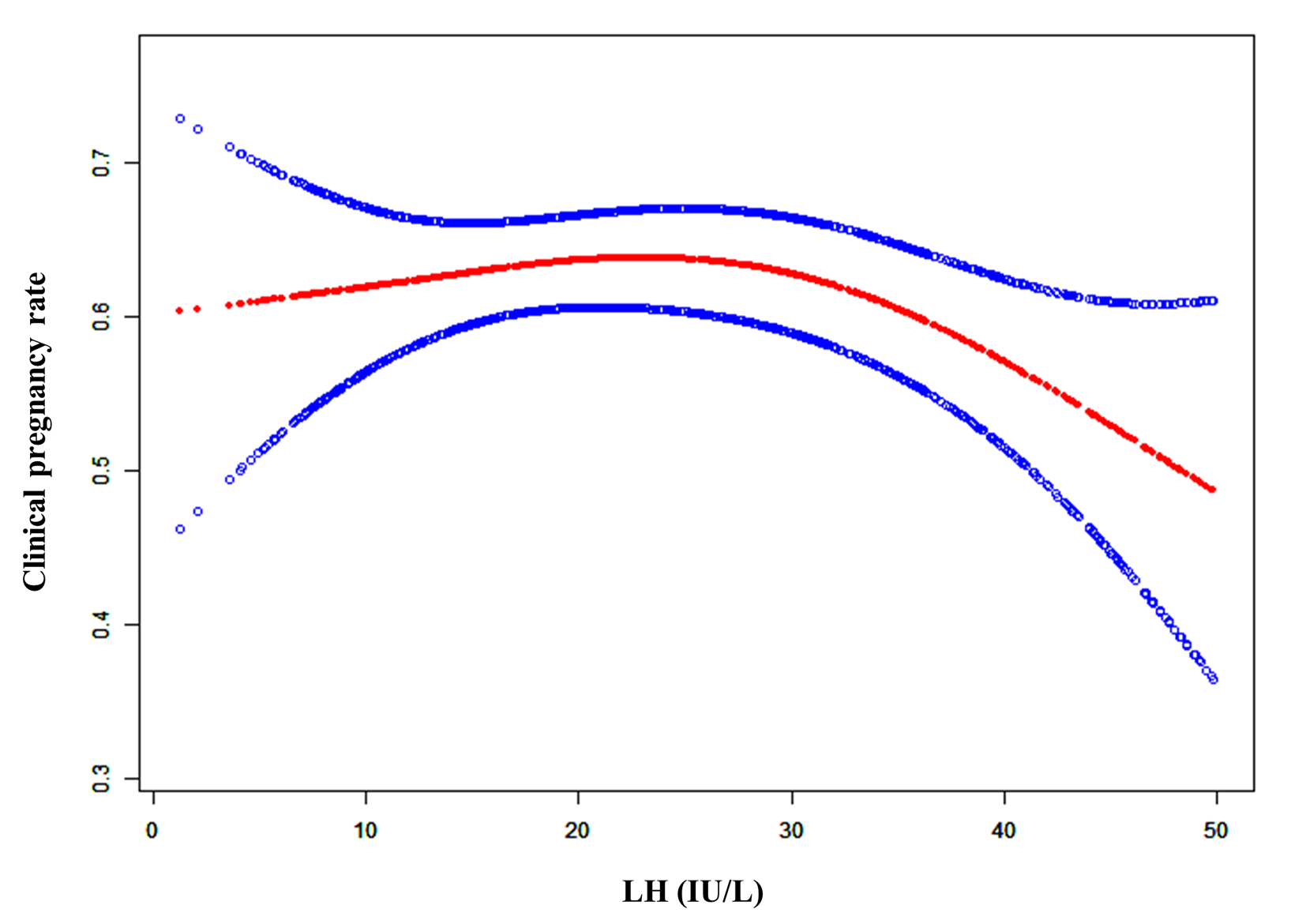

Supplement: Supplementary Figure 1 — A smooth fitting curve analysis of the relationship between LH levels on the hCG day and the clinical pregnancy rates. The clinical pregnancy rate of the patients decreased obviously as the LH level on the hCG day gradually increased when the LH level was beyond a certain value. [file Image_1.tif]

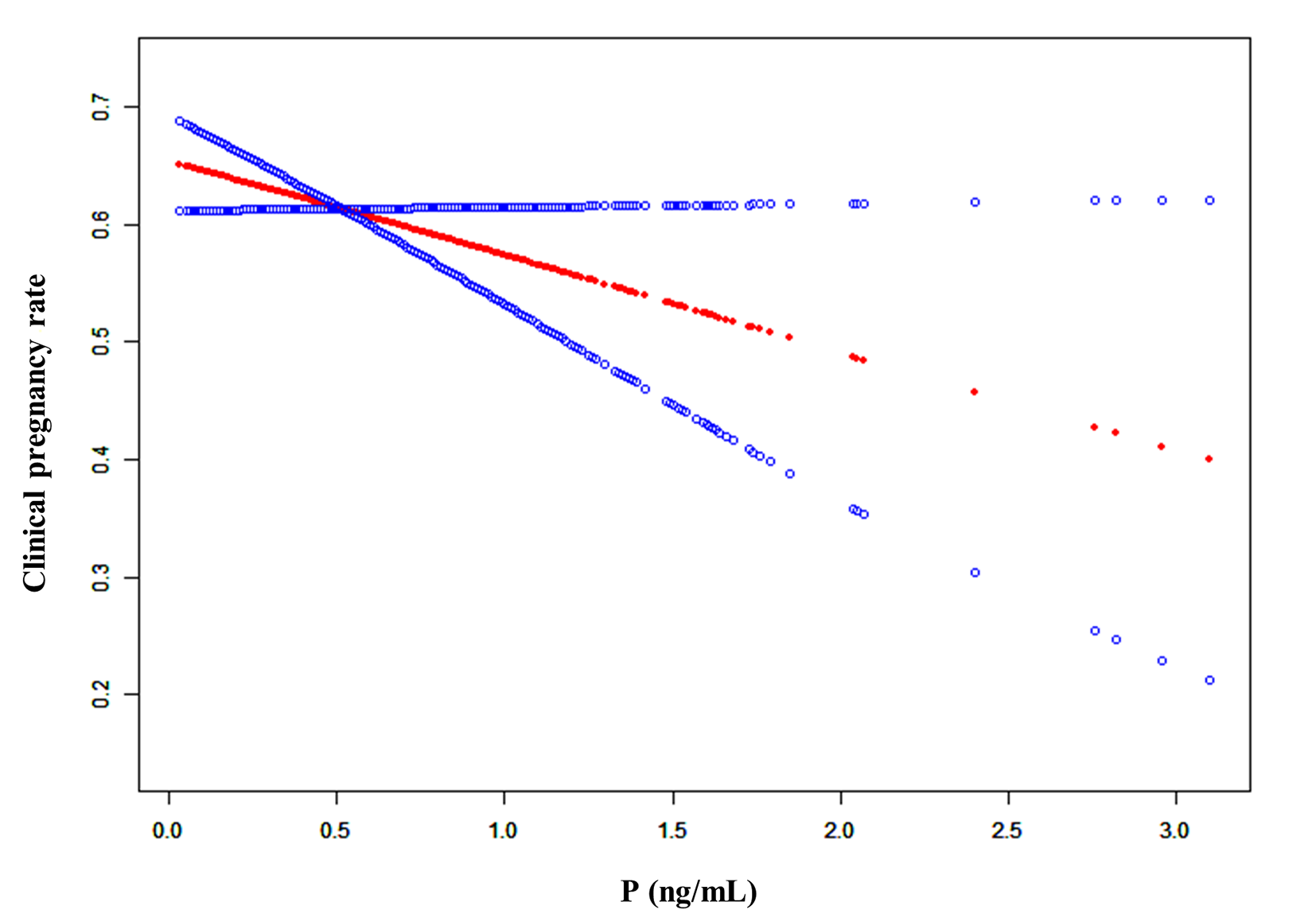

Supplement: Supplementary Figure 2 — A smooth fitting curve analysis of the relationship between P levels on the hCG day and the clinical pregnancy rates. The clinical pregnancy rate of the patients decreased obviously as the P level on the hCG day gradually increased. [file Image_2.tif]

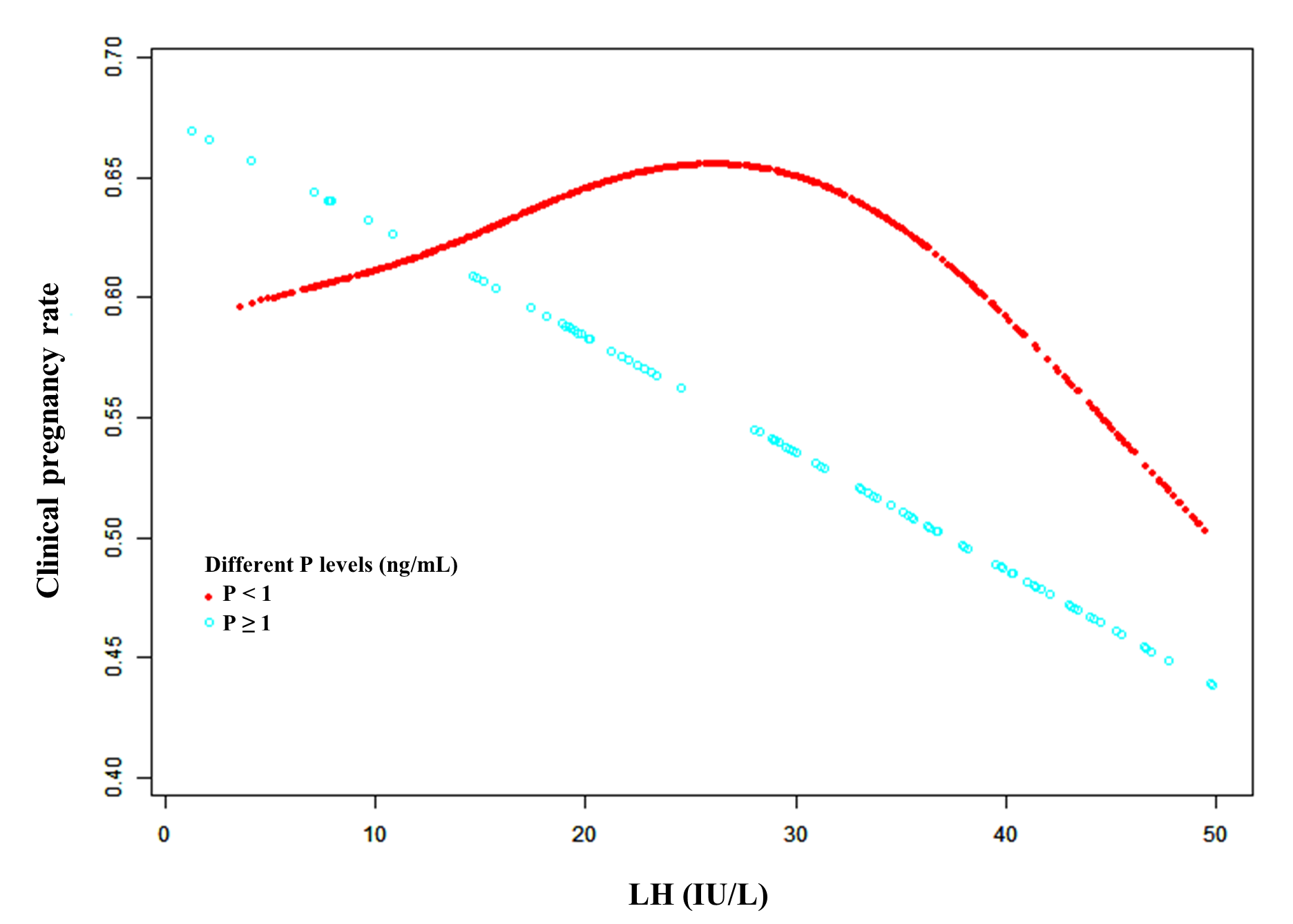

Supplement: Supplementary Figure 3 — A smooth fitting curve analysis of the relationship between LH levels on the hCG day and the clinical pregnancy rates of patients with different P levels. The clinical pregnancy rate decreased as the LH level on the hCG day gradually increased when the P level was equal to or greater than 1 ng/mL. In addition, the clinical pregnancy rate of the patients with a lower P level (less than 1 ng/mL) on the hCG day decreased as the LH level gradually increased when the LH level was beyond a certain value. [file Image_3.tif]
