# Supplementary material for: Adverse impact of elevated serum progesterone and luteinizing hormone levels on the hCG trigger day on clinical pregnancy outcomes of modified natural frozen-thawed embryo transfer cycles
Source: Front Endocrinol (Lausanne). 2022 Dec 1;13:1000047. doi: 10.3389/fendo.2022.1000047 (PMC9751419; doi:10.3389/fendo.2022.1000047)
Supplement: Supplementary file 4 [file Table_1.docx]

**Table S1.** General data of all enrolled patients and relevant data in mNC-FET cycles.

| **Female age (y)** | 31.52 ± 4.53 |
| --- | --- |
| **Male age (y)** | 32.84 ± 5.56 |
| **E_2_ on the hCG day (pg/mL)** | 307.50 ± 110.08 |
| **LH on the hCG day (IU/L)** | 24.35 ± 11.66 |
| **P on the hCG day (ng/mL)** | 0.51 ± 0.44 |
| **Em (mm)** | 10.19 ± 1.93 |
| **No. of transferred embryos (n)** | 1.40 ± 0.49 |
| **Type of transferred embryo** |  |
| **cleavage-stage embryo** | 40.23% (317/788) |
| **blastocyst** | 59.77% (471/788) |
| **Embryo implantation rate** | 52.81% (583/1104) |
| **CPR** | 61.29% (483/788) |
| **Early miscarriage rate** | 8.28% (40/483) |
| **LBR** | 53.81% (424/788) |

E_2_: oestrogen; LH: luteinizing hormone; P: progesterone; Em: endometrium thickness; CPR: clinical pregnancy rate; LBR: live birth rate.
