# Supplementary material for: Adverse impact of elevated serum progesterone and luteinizing hormone levels on the hCG trigger day on clinical pregnancy outcomes of modified natural frozen-thawed embryo transfer cycles
Source: Front Endocrinol (Lausanne). 2022 Dec 1;13:1000047. doi: 10.3389/fendo.2022.1000047 (PMC9751419; doi:10.3389/fendo.2022.1000047)
Supplement: Supplementary file 5 [file Table_2.docx]

**Table S2.** Effect modification of P levels on the hCG day on LBR according to different LH levels.

|  | **Group A:**  **LH < 32** | | | **Group B:**  **LH > 32** | | | **P for interaction** |
| --- | --- | --- | --- | --- | --- | --- | --- |
|  | **OR** | **95%CI** | **P value** | **OR** | **95%CI** | **P value** |  |
| **Crude** | 0.834 | 0.569-1.221 | 0.35 | 0.728 | 0.376-1.407 | 0.34 | 0.72 |
| **Adjusted** | 0.834 | 0.556-1.253 | 0.38 | 0.585 | 0.290-1.178 | 0.13 | 0.39 |

**Adjust for:** female age, male age, number of transferred embryos, type of transferred embryos and endometrial thickness.
